# cbpManager: a web application to streamline the integration of clinical and genomic data in cBioPortal to support the Molecular Tumor Board

**DOI:** 10.1186/s12911-021-01719-z

**Published:** 2021-12-20

**Authors:** Arsenij Ustjanzew, Alexander Desuki, Christoph Ritzel, Alina Corinna Dolezilek, Daniel-Christoph Wagner, Jan Christoph, Philipp Unberath, Thomas Kindler, Jörg Faber, Federico Marini, Torsten Panholzer, Claudia Paret

**Affiliations:** 1 Institute of Medical Biostatistics, Epidemiology and Informatics (IMBEI), University Medical Center of the Johannes Gutenberg-University Mainz, 55131 Mainz, Germany; 2 University Cancer Center (UCT), University Medical Center of the Johannes Gutenberg-University Mainz, 55131 Mainz, Germany; 3 Department of Hematology, Medical Oncology, and Pneumology, University Medical Center of the Johannes Gutenberg-University Mainz, 55131 Mainz, Germany; 4 Institute of Pathology, University Medical Center of the Johannes Gutenberg-University Mainz, 55131 Mainz, Germany; 5 Department of Medical Informatics, Friedrich-Alexander-Universität Erlangen-Nürnberg, 91058 Erlangen, Germany; 6 Junior Research Group (Bio-)medical Data Science, Faculty of Medicine, Martin-Luther-University Halle-Wittenberg, Halle, Germany; 7 Department of Pediatric Hematology/Oncology, Center for Pediatric and Adolescent Medicine, University Medical Center of the Johannes Gutenberg-University Mainz, 55131 Mainz, Germany

**Keywords:** Genomic data, Clinical data, Data management, Patient management, File generation, cBioPortal, Shiny, R, Bioconductor, Molecular Tumor Board

## Abstract

**Background:**

Extensive sequencing of tumor tissues has greatly improved our understanding of cancer biology over the past years. The integration of genomic and clinical data is increasingly used to select personalized therapies in dedicated tumor boards (Molecular Tumor Boards) or to identify patients for basket studies. Genomic alterations and clinical information can be stored, integrated and visualized in the open-access resource cBioPortal for Cancer Genomics. cBioPortal can be run as a local instance enabling storage and analysis of patient data in single institutions, in the respect of data privacy. However, uploading clinical input data and genetic aberrations requires the elaboration of multiple data files and specific data formats, which makes it difficult to integrate this system into clinical practice. To solve this problem, we developed cbpManager.

**Results:**

cbpManager is an R package providing a web-based interactive graphical user interface intended to facilitate the maintenance of mutations data and clinical data, including patient and sample information, as well as timeline data. cbpManager enables a large spectrum of researchers and physicians, regardless of their informatics skills to intuitively create data files ready for upload in cBioPortal for Cancer Genomics on a daily basis or in batch. Due to its modular structure based on R Shiny, further data formats such as copy number and fusion data can be covered in future versions. Further, we provide cbpManager as a containerized solution, enabling a straightforward large-scale deployment in clinical systems and secure access in combination with ShinyProxy. cbpManager is freely available via the Bioconductor project at https://bioconductor.org/packages/cbpManager/ under the AGPL-3 license. It is already used at six University Hospitals in Germany (Mainz, Gießen, Lübeck, Halle, Freiburg, and Marburg).

**Conclusion:**

In summary, our package cbpManager is currently a unique software solution in the workflow with cBioPortal for Cancer Genomics, to assist the user in the interactive generation and management of study files suited for the later upload in cBioPortal.

## Background

In recent years, advances in sequencing techniques have allowed a comprehensive detection of molecular aberrations within tumors in large studies, but also at the individual patient level. Such aberrations include mutations, copy number variations (CNVs), and change in gene expression and methylation, which can be visualized and analyzed via cBioPortal for Cancer Genomics [1, 2]. Currently, cBioPortal contains data from more than 180 studies, corresponding to approximately 48,000 tumor samples. Such data enable a deeper understanding of the tumor biology and support the development of novel therapies. One of the most important findings of this massive analysis of tumor samples is that molecular aberrations are not specific to tumor entities, but can be shared between different cancer types. This knowledge has led to a widespread application of off-label therapies in personalized protocols. Such therapies are discussed in special Molecular Tumor Boards (MTB) where experts from different fields collaborate with clinicians, to identify the best therapy for individual patients based on the molecular profile of the patient’s tumor [3]. The functional and clinical annotation of the identified aberration is a limiting factor in this process because multiple databases must be used to define the pathogenicity and “actionability “ of each molecular alteration. Indeed, while more than 1,000 genes are known to be involved in pathogenesis of cancer, only a small part can be therapeutically exploited [4]. Such information is already implemented in cBioPortal for Cancer Genomics, which enables the identified changes to be annotated automatically in order to simplify the prioritization of the reported hits and enhance their interpretation. In addition, cBioPortal also enables to collect and visualize the clinical data of the patient, including previous therapies, which are also required for the final selection of a personalized therapy. cBioPortal can be run as a local instance and could therefore be used in individual institutions for the documentation and interpretation of molecular aberrations in MTB. For this purpose, molecular data of single patients can be uploaded e.g. in the Mutation Annotation Format (MAF) and clinical data as plain text files with values separated by a delimiter. However, the creation of such files manually is time consuming and difficult to integrate into clinical practice. In order to improve the MTB workflow, facilitating the import of clinical and molecular data into a local instance of cBioPortal, we have developed the R package cbpManager.

## Implementation

### General design

In this publication, we use the nomenclature established by cBioportal. Accordingly, a cBioPortal study means a collection of patients. In a local cBioPortal instance, the provider can determine under which aspects patients are grouped into a study. For example, it makes sense to group all MTB patients of an Institution into one "MTB study". Another alternative is to group patients according to defined characteristics, such as the identical tumor entity.

**Fig. 1:**
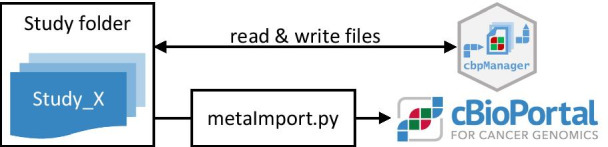
The study folder acts as a link between cbpManager and the local cBioPortal instance. cbpManager reads, generates and modifies study files stored in the study folder. The study upload to cBioPortal is an independent process of cbpManager and can be handled with the native script metaImport.py provided by cBioPortal

cbpManager is implemented using the R programming language and Shiny [5] framework. It can operate on already existing study files used by cBioPortal, but has no direct connection to a cBioPortal instance. The interface to cBioPortal is the directory where the individual study folders are stored—in the dockerized cBioportal version this folder is named "study". cbpManager operates on this folder in the sense that it can 1) read existing studies from this folder, 2) transform the data during an interactive session, and 3) save the transformed data back to this directory (Fig. 1). At the same time, this means that the upload process to cBioPortal is not managed by cbpManager and the generated study data has to be uploaded to the local cBioPortal instance independently from cbpManager, for example with the *metaImport.py* script offered by cBioPortal [6].

The main functionality of cbpManager can be accessed by a single call to the *cbpManager()* function, which starts the web application. As function arguments, the user passes the path to the study folder, and optionally inter alia a logging directory. During the user session, cbpManager lists the existing studies contained under the provided path as an interactive selection for the user with the possibility to edit them. A study directory for cBioPortal consists of two types of files—data files and metadata files. Each data file needs a metadata file that refers to it. When reading existing studies, cbpManager takes the files of a study as input and requires the data files to have specific names, in contrast to cBioPortal which determines the data file names based on the metadata files. When a study is to be loaded by cbpManger, the data files are read in, storing the information in a reactive object. This object is part of the reactive programming model used by Shiny, where the provided data can be interactively modified on the fly during a session. The user makes the interactive changes persistent by pressing the save buttons, which overwrites or creates the corresponding data and metadata files in the study directory.

The graphical user interface (GUI) layout is built using the shinydashboard package. The application is organized into several pages, which increases clarity and enables more flexible navigation during the editing workflow (see section “Results”). The individual pages correspond to a subset of the different data types that cBioPortal can process as input. The study data of the individual pages is represented in interactive tables generated with the package DT [7]. The tables can be edited through a number of functional buttons and modal dialogs, which are also based on Shiny's reactive programming model. Many of the buttons are designed in a modular manner, allowing effective maintenance and customization of functionality.

In addition, cbpManager offers validation of the currently loaded study using the cBioPortal validator. This requires Python and several other dependencies. cbpManager installs via the R packages reticulate [8] and basilisk [9] either during the session or alternatively with the command *setupConda_cbpManager()* a conda environment with Python and the necessary dependencies. The execution of the script before the actual *cbpManager()* call has the advantage to avoid longer waiting times during the session.

The functionality of cbpManager is comprehensively described in the package vignette and can be browsed at https://arsenij-ust.github.io/cbpManager/index.html, built with the pkgdown [10] package. cbpManager leverages bootstrap components (e.g. tooltips, collapsible elements) as provided via the shinyBS [11] package to provide in-app documentation of the functionality. This is also showcased in interactive tours, based on rintrojs [12], that can be taken to gain familiarity with the user interface while performing common tasks.

cbpManager has been tested on macOS, Linux, and Windows. cbpManager is freely available via the Bioconductor project [13], and its development version can be found at https://github.com/arsenij-ust/cbpManager/.

### Deployment in a clinical environment

**Fig. 2:**
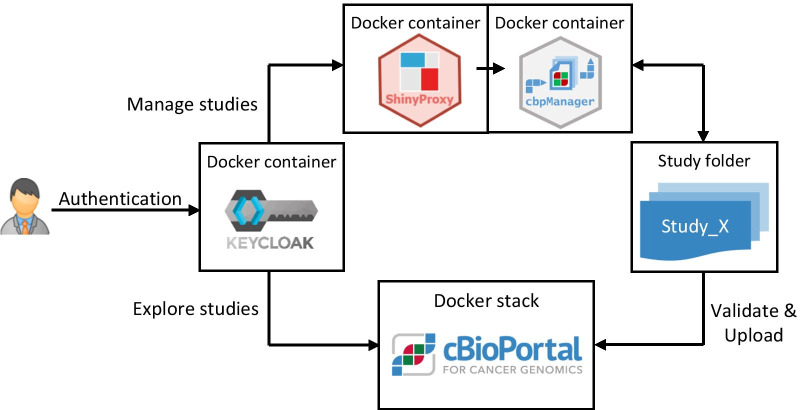
IT-Architecture of dockerized services enhancing security. The user authenticates via Keycloak and is redirected to the Docker-container of cbpManager via ShinyProxy. Behind the scene, the study files are stored in the respective subfolders of the study directory. The studies are uploaded to cBioPortal via automated upload processes. cBioPortal provides mechanisms for handling user and group rights and supports Keycloak as Identity Provider

Since cbpManager is an R package, anyone with little R knowledge can install cbpManager locally in their R environment and try out the application in advance. For this purpose, the cbpManager provides a simple test study right out of the box, with the help of which the user can get to know and experiment with the use of the cbpManager. However, in the use case of an MTB, it is essential that several users can access the same study data in the cbpManager and that a certain degree of security is ensured. To meet these requirements, we containerized the cbpManager and deployed it with ShinyProxy. In a typical use case, a service facility provides a dockerized instance of cbpManager to documentalists and physicians for interactive documentation and management of patient data. ShinyProxy launches a Docker container each time a user runs the application, providing an isolated environment for that session. The respective container is automatically removed by ShinyProxy after the user has exited the session. This allows multiple users to access the same data with the cbpManager and operate cooperatively on the same studies. To meet the increased security standards of clinical environments, an authentication of the cbpManager application can be realized with an authentication server (such as Keycloak or LDAP) linked to ShinyProxy (Fig. 2). Along this line, we provided a gitlab repository at https://gitlab.miracum.org/arsenij_temp/cbpmanager.deploy containing Dockerfiles for cbpManager, and ShinyProxy with an exemplary authentication configuration. To facilitate deployment, we created a Docker Compose file that allows both services (cbpManager and ShinyProxy) to be created and started with one command.

The speed and performance of cbpManager will vary depending on the hardware specifications available. Since cbpManager does not execute any calculation-heavy processes, it can be run on computers/ servers with average specifications, e.g. 8 Gb RAM, Intel(R) Core i5 @ 2.60 GHz with 2 cores and 500 Gb HDD. The RAM during a session depends on the size of loaded studies. Even if a large study is loaded, e.g. with about 24,000 samples and patients, the RAM usage is below 100 Mb—as measured with the profvis package.

## Results

### Workflow of cbpManager

**Fig. 3:**
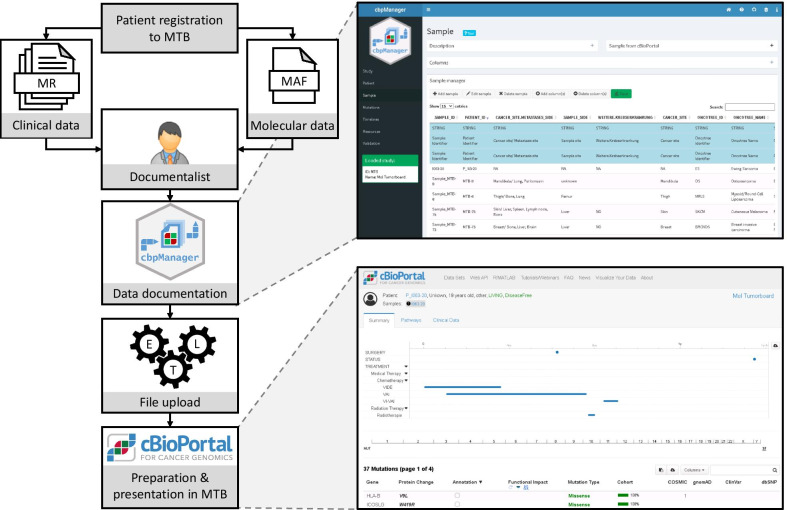
Role of cbpManager in use case of a MTB. For the discussion of a patient in MTB, a molecular analysis of the tumor is performed and clinical information such as diagnosis and previous therapies are retrieved from the medical records (MR). A medical documentalist applies cbpManager to create data files suitable for the upload in cBioPortal. For this, molecular data should be prepared in a MAF format, while samples and clinical data are added via the cpbManager interface. Once imported in cBioPortal, molecular aberrations are automatically annotated and previous therapies are visualized. This facilitates the patient-specific selection of a therapy in MTB

In the context of an MTB use-case, cbpManager serves as a tool to manage and prepare clinical and molecular data for upload to cBioPortal, where new patients need to be added continuously and new data is generated during the course of treatment. Below we propose a workflow where the cbpManager is incorporated into the procedure of an MTB. Figure 3 shows the workflow starting with patient registration for the MTB. Clinical data, e.g. the patient's master data with the previous course of therapy, and data from the molecular diagnostic analysis are made available to a documentalist. It is worth mentioning that these data are site-specific, e.g. in digital structured or unstructured form or in paper form. The documentalist adds the new patient with all his data to the MTB study in cbpManager. In the background, cbpManager creates the necessary files in cBioPortal compliant format. These files are uploaded by the IT administrators or by automated ETL processes (see section “IT-infrastructure around the cbpManager”) to the hospital's cBioPortal instance, where they are available to the interdisciplinary MTB team for preparation and presentation in the MTB.

To date, cbpManager has supported six data types defined by cBioPortal: Cancer Study, Patient Data, Sample Data, Mutation Data, Timeline Data, and Resource Data. cbpManager is capable of reading and producing the data and meta files of these data types. Each data type is featured in a dedicated page of cbpManager.

**Fig. 4:**
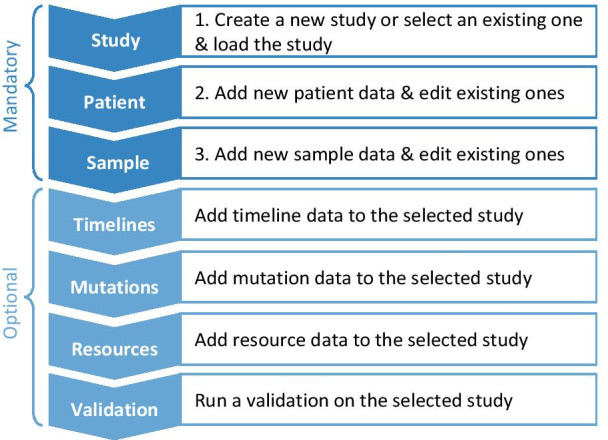
Common workflow for the use of the cbpManager. The blue arrows symbolize the individual pages of the cbpManager from top to bottom. The text boxes explain the function of the respective page. At the beginning, there are the pages that have to be edited compulsorily. Afterwards, the pages follow, which can be supplemented depending upon existing data

**Fig. 5:**
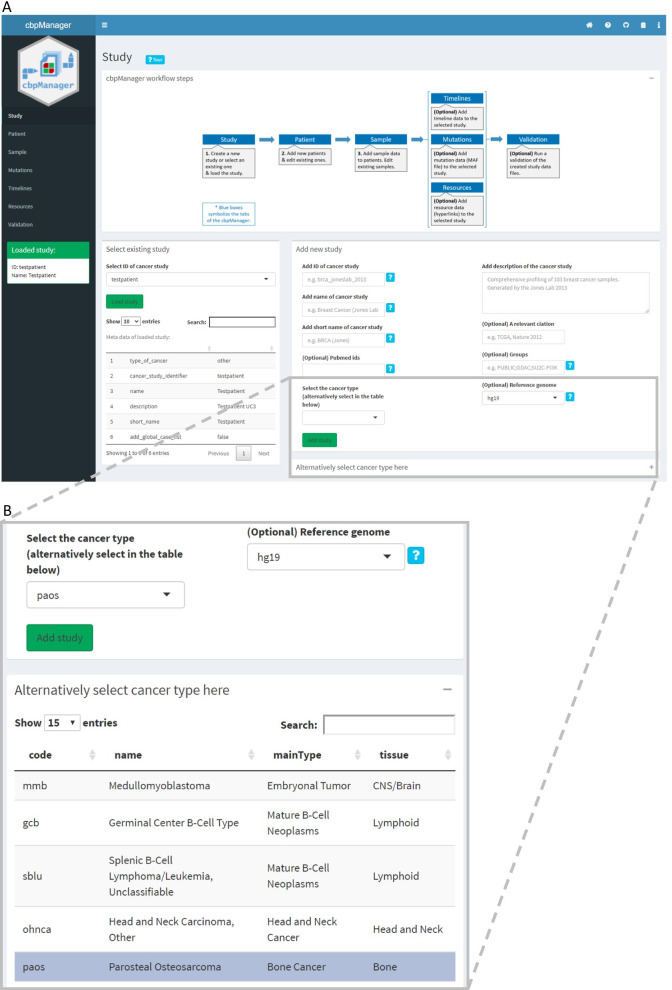
*Study* page of cbpManager. **A** An overview over cbpManager’s first page allowing the user to create new studies or select and load existing ones. The text fields for the addition of a new study represent meta information of the study and are later written to the meta study file. **B** A close up of the page showing the OncoTree table used for the cancer type selection

In order to minimize the occurrence of mistakes during the use and to correctly assign new entries to a patient, a certain sequence of operations should be followed in the workflow of the cbpManager (Fig. 4). Thus, a new study has to be created or an existing one selected before adding Patient or Sample Data. Since a link between patients and samples or patients and timeline entries is ensured via a patient ID, it is necessary to first create a new patient on the *Patient* page before adding new Sample or Timeline Data to this patient. This order is implicitly given by the arrangement of the menu items, as well as by programmatic error catching, and explicitly by a graphic on the first page of the cbpManager, the vignette, and an interactive tour (Fig. 5A). Optional steps, such as the integration of molecular data and timelines can be taken after completing the mandatory operations of the cbpManager workflow.

### Study page of cbpManager for creation and loading of studies

The homepage of cbpManager is the *Study* page, where the user can either create a new study or select from existing studies to load and edit (Fig. 5A). The *Add new study* panel located on the right side reflects parameters known in cBioPortal as meta-study file, which contains metadata such as cancer study identifier or the description of the cancer study. The cancer type has to be written down as an abbreviation. To make this easier for the user, the expandable panel below provides an interactive table based on OncoTree [14], where the user can more easily find the cancer type by using search and sort functionality. In this OncoTree table, the user can then select the cancer type by clicking on the row and the respective dropdown menu *Select the cancer type* will be synchronized with it (Fig. 5B). In case of studies with multiple cancer types—as it often occurs in the context of MTBs—the type can be specified as *mixed*. After clicking the *Add study* button, the study will be created in a new sub folder under the defined study directory and the respective meta-study file will be generated. This folder is named after the input value of the *Add ID of cancer study* text field. Metadata of a study can be changed retrospectively. In order to further manage a study, it has to be loaded by selecting the respective study in the dropdown menu *Select ID of cancer study* on the left panel. After pressing the *Load study* button, a table containing the metadata below the button as well as a green box in the navigation sidebar appear signaling the user about the successful data upload.

### Managing patient data in cbpManager

**Fig. 6:**
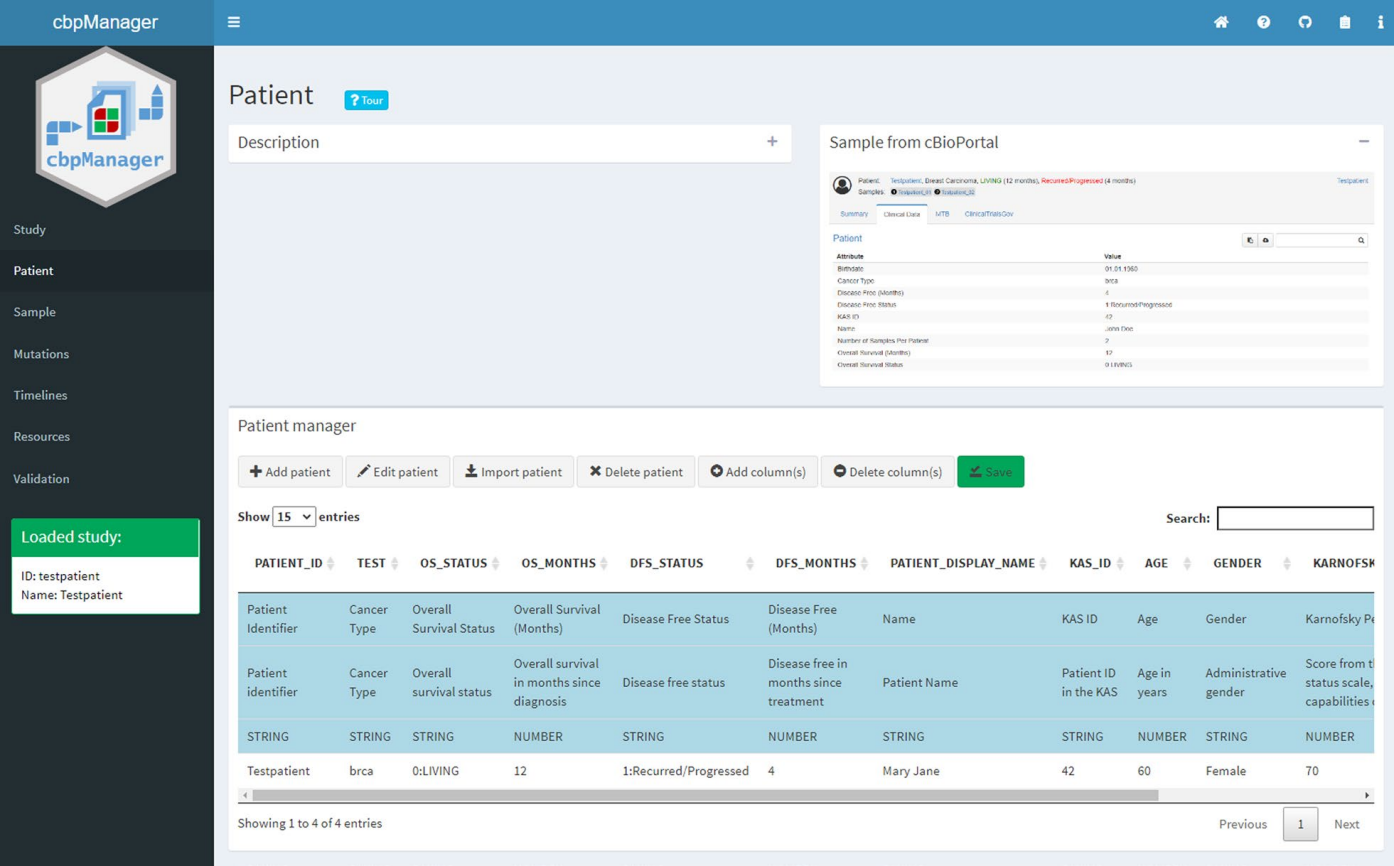
*Patient* page of the cbpManager representing the Patient Data of the example study *Testpatient* provided with the cbpManager. Three main panels contain a description, an exemplary representation of how the data is displayed in cBioPortal (Sample from cBioPortal), and the table representing Patient Data of the currently loaded study, accompanied by several buttons that allow editing the table. Only the column *PATIENT_ID* is mandatory, the remaining optional columns are a mix of pre-defined and customized ones

Once a new study has been created or an existing one has been selected, new patients can be added by using the patient manager on cbpManager’s *Patient* page (Fig. 6). In the upper area, it has a *Description* box containing important information on filling in the Patient Data as well as instructions for handling, and a *Sample from cBioPortal* box with an exemplary representation of the Patient Data in cBioPortal (both boxes are collapsed in the following image for better clarity). The *Patient manager* panel contains several function buttons and a central table with the patient information. The first three light blue lines must contain a short name, a long name, and the data type of a column. These rows are required for converting the table to the cBioPortal format and contain metadata of the single attributes (columns) that are later used by cBioPortal for the representation of the attributes. Each further row represents a patient.

**Fig. 7:**
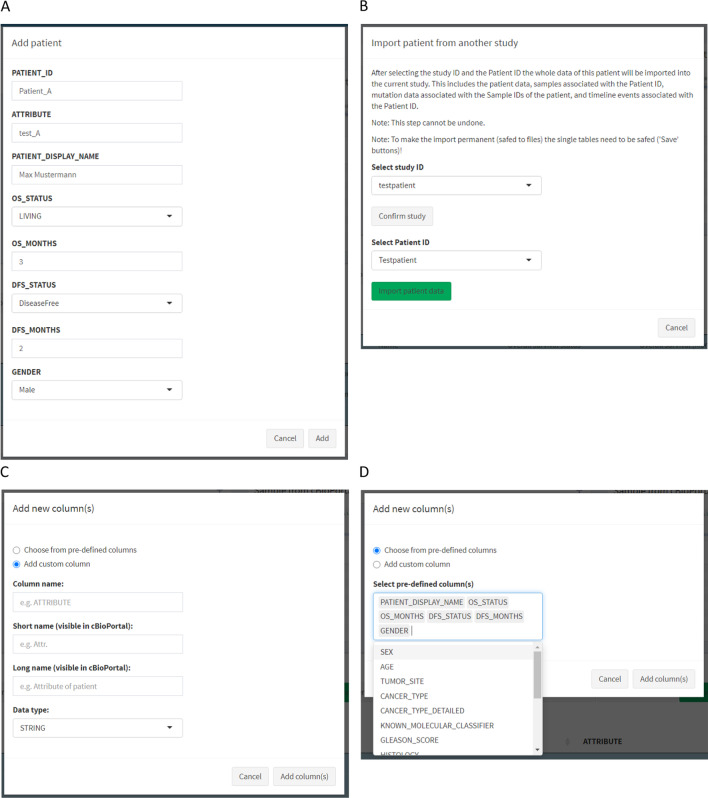
Exemplary dialog windows. **A** Dialog window triggered by the button *Add patient* showing an input field per existing attribute in the *Patient manager* table enables the user to add a new row with patient data. **B** Dialog window triggered by the button *Import patient* enables the import of patients from other studies by importing data of the selected patients into the currently loaded study. **C** Dialog window triggered by the button *Add column(s)* provides the user the possibility to add a custom column to the *Patient manager* table. A custom column requires a column name, a short name, a long name, and the data type of the column. The data type can be numeric, boolean, or a character string. **D** The dialog window triggered by the button *Add column(s)* alternatively enables the user to add predefined columns to the *Patient manager* table

The table can be modified using the upper row of buttons. The following functionalities are available: *Add patient*, *Edit patient*, *Import patient*, *Delete patient*, *Add column*, *Delete column*, and *Save*. The *Add patient* button opens a dialog box containing one entry field per existing column (Fig. 7A). For some predefined attributes the input fields are specific, e.g. only numeric values for the predefined attribute *OS_STATUS*. The input field *PATIENT_ID* is the only mandatory one. After confirming the input, the values are transferred to the table of the *Patient manager* panel. It is possible to import Patient Data from another existing study with the *Import patient* button. In the respective dialog box the study has to be selected first and then the patient ID (Fig. 7B). During the import, not only Patient Data is imported, but also Sample, Mutation, and Timeline Data of the respective patient are entered. The *Add column(s)* button allows the user to add a new column to the table. In the appearing dialog window one can choose from two options 1) Add a user-defined column (Fig. 7C), and 2) Choose from predefined columns (Fig. 7D). While in the first case the user has to specify the metadata of the column in addition to the column name, such as a short and long name of the attribute, in the case of predefined attributes this metadata is already set internally, and is added to the table automatically. For the predefined attributes, we followed the guidelines of cBioPortal, some of the attributes are used in cBioPortal for survival plots, additional information of the patient description in the header, the pan-cancer summary statistic tab, or other specific functionalities. As it is the case with all *Save* buttons of the cbpManager, it is necessary that the user makes the changes permanent by pressing the button after editing a table. This results in the corresponding data and meta files being created—or overwritten if they already exist.

### Adding and editing samples of a cancer study in cbpManager

The *Sample* page has the same structure as the Patient page, since the format of the files resulting from the two pages does not differ. The few functional differences between the two pages are that the rows now represent samples, there is no button for patient import, the *PATIENT_ID* and *SAMPLE_ID* columns are mandatory when creating a new sample, and sample-specific columns are listed among the predefined columns.

### Adding genomic informations

This version of cbpManager allows adding only mutation information. The *Mutations* page offers the user the possibility to upload MAF files and previews existing Mutation data of the currently loaded study as a data table. If mutation data is already available, the content of the uploaded MAF file will be concatenated to the already existing data.

The MAF file has to meet certain requirements defined by cBioPortal, i.e. contain at least the columns *Hugo_Symbol*, *Tumor_Sample_Barcode*, *Variant_Classification*, and *HGVSp_Short* as defined in the chapter “Formats” of the cBioPortal documentation [15]. If the user does not have any mutation data, he must use the *Save MAF file* button to generate a corresponding empty data file with associated meta files so that cBioPortal does not throw an error during upload.

### Managing timeline data by editing predefined and custom timeline tracks

**Fig. 8:**
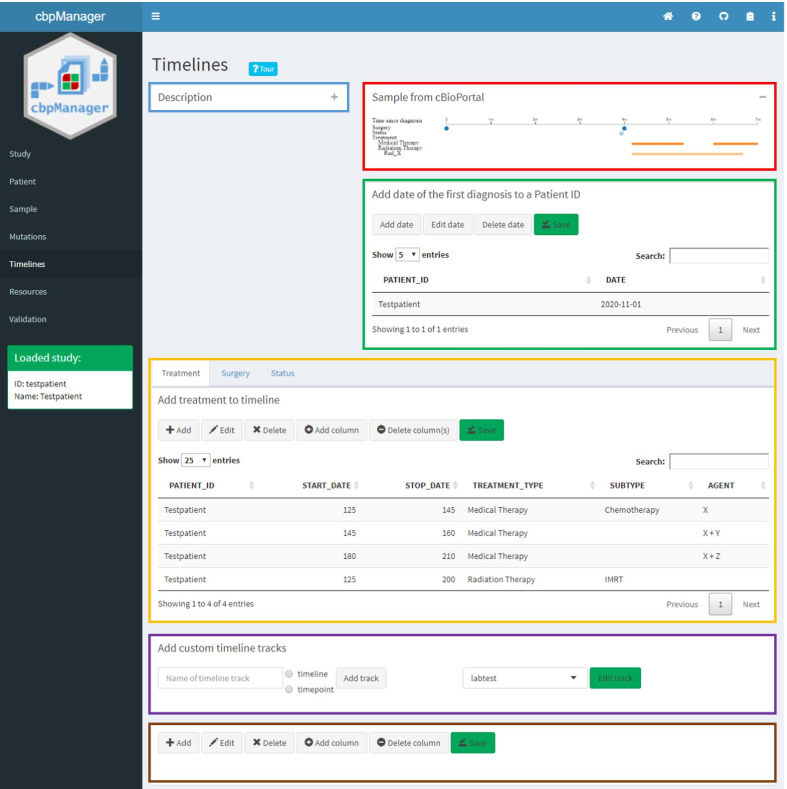
*Timeline* page of the cbpManager representing the Timeline Data. The page is divided in six panels containing 1) a description (collapsed panel—blue box), 2) an exemplary image of the cBioPortal representation (red box), 3) a panel for managing dates of first diagnosis (green box), 4) a panel containing tab separated timeline track manager (yellow box), 5) a panel for the addition and selection of further timeline tracks (violet box), and 6) a panel where the table of the selected custom timeline track is shown (brown box)

The *Timelines* page allows the user to enter information about previous therapies and the patient status which are displayed as a timeline graph in cBioPortal. It enables editing the timeline tracks *Treatment*, *Surgery* and *Status*. Furthermore, it is possible to create and edit user-defined timeline tracks (Fig. 8). Since cBioPortal represents the number of days starting from the initial diagnosis point instead of a real date for the start or end of a timeline event, one would have to specify the initial diagnosis date for each event as well. This is repetitive and time-consuming in total, which is why we decided to create a patient first diagnosis date table as an intermediate step. We solved this issue by forcing the user to assign a diagnosis date to the patient once, before adding timeline events to a timeline track. This action takes place in the panel *Add date of the first diagnosis to a Patient ID*. cbpManager uses the date of the first diagnosis to later calculate the number of days for each timeline event of the corresponding patient ID.

To create custom timeline tracks, the user has to specify the name of the timeline track first, and then define whether the event has a time span (timeline) or is a point event (timepoint). This is relevant because timeline events have a start and stop date, while timepoint events only need to have a start date. To edit a timeline track, it needs to be selected from the dropdown menu and confirmed by clicking on the *Edit track* button. The data of the timeline track will be displayed in the panel below. The editorial functionalities are the same as for the rest of the pages. Again, each change must be saved with the *Save* button to make it persistent.

### Additional information

In the *Resources* page additional documents can be added to patients, samples, and studies. A resource describes any available web page, pdf, txt, png, or json file and is represented in cBioPortal as a hyperlink and a tab containing the web based resource. This allows the physician to add pdf based reports or important web pages to samples, patients, or the whole study. The data structure defined by cBioPortal to add a resource is not very intuitive. cbpManager enhances the understanding for the user by the arrangement of the different panels, and through recurring functionalities.

### Final validation of created study files

The *Validation* page allows the user to quickly validate the created or modified study files. The validation checks whether the study is suitable for the upload in cBioPortal. In the background cBioPortal’s *validateData.py* script was used to generate the html validation report, which is then inserted in the cbpManager GUI. The downloadable report generation is triggered by the *Validate* button. Afterwards, the report can be downloaded. The report provides information about which of the study files are present, which files cause warnings or errors and thus prevent the upload to cBioPortal, and usually a precise description of the warnings and errors.

## Discussion

The exploitation of molecular data in patient management is an increasing need in the era of biomarker-driven medicine. However, the use of such data requires technical knowledge and infrastructure which are not always available in hospitals. Even more complex is to connect clinical and molecular data of a patient. While cBioPortal allows the storage, visualization and annotation of molecular data and the storage and visualization of clinical data, its use in hospitals is made difficult by the different steps required for the import of all necessary information. This problem is solved by our proposal cbpManager.

### IT-infrastructure around the cbpManager

The simplest way to make cbpManager available to multiple users is to use the application in a containerized environment as described above in section “Deployment in a clinical environment”. Such containerized applications are becoming the preferred deployment vehicle for micro services in healthcare. Installing cbpManager as a Docker container enables portable and reproducible deployment.

The intuitive interface of cbpManager allows the import of patients’ molecular data without any computational background. However, mutations are retrieved from bioinformatics best practice workflows [16] analyzing patients’ sequencing data. Variant calling algorithms inside of these workflows often use the variant call format (VCF) as state-of-the-art file format to store their immediate results. It contains a header section with metadata and a body section with eight mandatory columns, such as chromosome, position, reference allele, alternative allele and some quality statistics, but it can easily be extended with any number of additional information by further columns. cBioPortal however uses the MAF file format. MAF differs from VCF in that it is a tabular format without a header section and requires different obligatory columns (*Hugo_Symbol*, *Variant_Classification*, *Variant_Type*), which have to be annotated first. To convert a VCF to a MAF file there are several options, e.g. the vcf2maf tool [17] uses the annotation generated by the Variant Effect Predictor (VEP) [18] and converts it to a MAF file. Alternatively, ANNOVAR [19] can be used in combination with the R package maftools [20]. Also, the recent version of GATK [21] provides an option to output in MAF.

cbpManager is currently unique in its functionality. Thus, facility-specific solutions are an alternative to preparing the data for the cBioPortal import. Such solutions may be automated ETL routes to clinical information systems but require to be developed and implemented at the respective sites. The study import can also be designed variably. At this point, there is either the cBioPortal python script *metaImport.py* [6], which can be executed automatically on a regular basis or after certain triggers, or the dockerized application cbioportal-staging [22], which can extract files from certain locations, transform, validate and load them into cBioPortal. The latter application has various useful features and focuses mainly on the automated data upload, but does not replace the GUI offered by cbpManager and the possibility to flexibly edit data of individual patients in a tabular manner. At the same time, cbpManager does not replace these applications because it lacks the functionality to load study data into cBioPortal.

In many cases, the facility already has existing patient documentation in the form of registers, databases or Excel spreadsheets. The cbpManager is also only suitable to a limited extent for the initial import of this patient base into cBioPortal, as patients have to be inserted individually and this would be time-consuming. While the efforts for creating a generalized solution might be too extensive, given that facilities often adopt unique combinations of content and its format, we still recommend the usage of custom scripts to streamline operations for the initial and one-time preparation of a broad patient base in the appropriate cBioPortal data formats.

### Information supported by the current version of cbpManager

cbpManager allows importing clinical information available via the documentation system of a hospital. Minimum information is already implemented as default, but the system allows the user to add customised information in a simple and intuitive manner. The current version of cbpManager uses OncoTree for definition of the tumor entity. The OncoTree is an open-source ontology that was developed at Memorial Sloan Kettering Cancer Center (MSK) for standardizing cancer type diagnosis [14]. This ontology is not currently the standard used by pathologists which generally define tumor entities based on the WHO criteria. The implementation of WHO criteria and definition in further versions of cbpManager is possible. Concerning molecular data, this version of cbpManager allows importing only mutations. Mutations are currently, together with some fusions, the most discussed molecular aberrations in a MTB. cBioPortal allows other data formats such as copy number variations, fusions, methylation, protein and RNA expression data that are more and more available via omics analysis of single patients. Moreover, such data formats could be exploited to import information generally available via standard diagnostic procedures. For example, the Fusion Data format could be used not only to import fusions detected by RNA-Sequencing but also translocations detected by FISH (fluorescent in situ hybridization). The expression or protein data format could be used to document immunohistochemistry results.

### Deployment of the cbpManager at several clinical centres in Germany

We developed cbpManager principally to support the MTB documentation workflow in the frame of the Use Case “From Knowledge to Action—Support for Molecular Tumor Boards” of the MIRACUM consortium (Medical Informatics in Research and Care in University Medicine [23]), which focuses on the provision of IT and bioinformatics support for exploitation and visualization of data required in a MTB [24]. In addition, it is already being used prototypically via Halle in the SMITH consortium [25], via Lübeck in HiGHmed [26], and prospectively via the Bavarian Center for Cancer Research (BZKF) in locations of the DIFUTURE consortium [27].

### Outlook

We are currently working on the further development of cbpManager. In the next versions of cbpManager, we aim to cover more file formats of cBioPortal, e.g. copy-number alteration data, fusion, and expression data, since such complementary information is increasingly available in routine clinical activities and are gaining more importance in the therapy recommendation process. A useful addition would be the management of user roles and rights for viewing and editing individual studies and even patients. This is relevant in the clinical setting because it may often be the case that the people who have access to the cbpManager are not allowed to view all studies or individual patients due to data protection restrictions. We will implement usability feedback and clinical documentation standards as far as possible.

As mentioned above, cbpManager was developed in the framework of the MIRACUM use case to support MTBs. Among other things, this use case focuses on the extension of cBioPortal functionalities. Like other tools in this use case, cbpManager will be continuously developed and optimized over the duration of the use case. This ensures that cbpManager is adapted to possible major updates of cBioPortal. Generally, cbpManager is expected to be compatible with cBioPortal in the long run, as changes to file formats and data types have been rare so far and were mostly backward compatible with previous file formats. If cBioPortal introduces updates concerning file formats, the programmatic adaptation of cbpManager would likely not be a major hurdle, as only the read-in and output functions would have to be adapted.

## Conclusions

Because of cbpManager’s interactive web-based GUI, it is an intuitive application for the creation of cBioPortal-compatible files containing center-specific molecular and clinical data. It has the potential to improve the MTB workflow by simplifying the laborious process of entering and validating patients’ data and preventing diverse errors before they occur during the cBioPortal upload. The docker-based installation makes cbpManager easy to integrate in complex IT-infrastructure and allows secure access to the application in clinical environments. We anticipate that cbpManager could facilitate the incorporation of MTBs into standard-of-care oncology practices.

## Availability and requirements

Project name: cbpManager. Project home page: https://bioconductor.org/packages/cbpManager/ (release) and https://github.com/arsenij-ust/cbpManager/ (development version). Project documentation: rendered at https://arsenij-ust.github.io/cbpManager/. Operating system(s): Linux, Mac OS, Windows. Programming language: R. Other requirements: R version 4.1 or higher, Bioconductor release 3.13 or higher. License: AGPL-3. Any restrictions to use by non-academics: none.

## Data Availability

Data sharing is not applicable to this article as no datasets were generated or analyzed during the current study.

## Abbreviations

CNV: Copy number variations
FISH: Fluorescent in situ hybridization
GUI: Graphical user interface
MAF: Mutation Annotation Format
MIRACUM: Medical Informatics in Research and Care in University Medicine
MTB: Molecular Tumor Board
VCF: Variant call format
